# Nux Vomica in Neuralgia

**Published:** 1844-07-27

**Authors:** 


NUX VOMICA IN NEURALGIA.

*M. Roclants*, a Dutch physician, reports most favorably of the effects of this potent drug in severe cases of neuralgia of the face and other parts, and communicates at the same time the therapeutic results obtained by many of his professional friends. Out of 29 severe cases, a perfect cure was effected in 25, and decided relief was afforded in the other four. The dose, in which the powdered nux vomica was administered, was from three to ten grains, and upwards, in the course of the twenty-four hours. In all cases its effects should be narrowly watched, as unpleasant consequences have occasionally resulted from incaution on the part of the physician. *M. Roclants* is inclined to regard the nux vomica as, on the whole, the most efficient and certain remedy against severe neuralgia; he has seen several cases, which had resisted the prolonged administration of steel, bark, and all other most approved means, yield to its use.—*Med. Chir. Rev*

